# The effects of color and neutral density filters on dynamic accommodation in individuals with mild traumatic brain injury

**DOI:** 10.3389/fnins.2025.1630514

**Published:** 2025-08-11

**Authors:** Nawaf M. Almutairi, John Hayes, Yu-Chi Tai, Khawlah Alfaifi, Mohammed M. Alnawmasi, Muteb K. Alanazi, Bandar M. Alenezi, Sulaiman Aldakhil, Chunming Liu

**Affiliations:** ^1^Department of Optometry, College of Applied Medical Sciences, Qassim University, Buraydah, Saudi Arabia; ^2^College of Optometry, Pacific University, Forest Grove, OR, United States; ^3^College of Biomedical and Life Sciences, School of Optometry and Vision Sciences, Cardiff University, Cardiff, United Kingdom; ^4^Department of Optometry, College of Applied Medical Sciences, King Saud University, Riyadh, Saudi Arabia

**Keywords:** dynamic accommodation, mild traumatic brain injury (mTBI), neutral density filter, color filter, accommodative dysfunction

## Abstract

**Introduction:**

Visual symptoms related to accommodation are frequently reported after mild traumatic brain injury (mTBI), yet the effect of spectral and luminance-altering filters on dynamic accommodative performance remains unclear. This study objectively measured dynamic accommodation in individuals with mTBI and healthy controls under various filter conditions to determine if visual performance could be improved.

**Methods:**

Thirty participants with a medically diagnosed history of mTBI (age range 18–33 years) and 54 healthy controls (age range 21–30 years) completed monocular dynamic accommodation testing under three randomized viewing conditions: baseline with no filter (NF), a subjectively selected color filter (CF), and a luminance-matched neutral density filter (ND). Accommodation and disaccommodation responses to 5.00 D step stimuli were continuously recorded at 50 Hz using the PowerRef 3 photorefractor. First-order response parameters were extracted, such as peak velocity, response amplitude, latency, and response time. A mixed linear model was used to assess group, filter, and interaction effects.

**Results:**

At baseline (NF), the mTBI group showed significantly reduced accommodation peak velocity (mean difference = −1.68 D/sec) and response amplitude (mean difference = 0.55 D) compared to controls (*P* < 0.05). CFs did not significantly alter any response parameters in either group. In contrast, ND filters significantly increased accommodation peak velocity (mean difference = +1.77 D/sec) and amplitude (mean difference = 0.67 D) in the mTBI group (*P* < 0.001). Latency and response time remained stable across all conditions and groups.

**Discussion:**

Under baseline circumstances, especially in speed and magnitude of response, participants with mTBI showed apparent deficits in dynamic accommodation. These findings indicate that, rather than spectral filtering, brightness modulation via ND filters can significantly enhance accommodative performance in individuals with mTBI. This suggests ND filters may serve as a viable clinical intervention for improving accommodative dynamics in this population.

## Introduction

Traumatic brain injury (TBI) affects 2.8 million people annually in the United States and poses significant public health concerns (Peterson et al., 2019). Mild TBI (mTBI) accounts for about 75% of all reported cases [Faul et al., 2010; Control (US) NC for IP, 2003]. With the widespread impact of mTBI on neurological, cognitive (Wolf and Koch, 2016) and physical functions, the visual system is commonly affected (Merezhinskaya et al., 2019). Mild TBI frequently results in considerable visual impairments, especially in accommodation (Green et al., 2010; Thiagarajan and Ciuffreda, 2014; Dutta et al., 2024; Chen et al., 2020; Wiecek et al., 2021; Haensel et al., 2024; Almutairi et al., 2025) and vergence eye movements (Mani et al., 2018), which are crucial for maintaining clear vision (Merezhinskaya et al., 2019). Individuals with mTBI commonly experience accommodative insufficiency and infacility (Capó-Aponte et al., 2012; Master et al., 2016), increased lag, decreased peak velocity, and increased latency (Green et al., 2010; Thiagarajan and Ciuffreda, 2014), as well as alterations in accommodation microfluctuations (Almutairi et al., 2025). These impairments can affect their ability to perform everyday visual tasks such as reading. Given that clear vision is critical to daily functioning, understanding how mTBI impacts accommodation is essential.

The accommodation system modifies the eye's refractive properties, enabling the retinal image to achieve fine focus (Read et al., 2024). Numerous studies have examined the dynamic accommodation characteristics of the human eye (Sun and Stark, 1986; Heron et al., 2001; Ciuffreda and Kruger, 1988). The primary function of this dynamic reaction is to modify the eyes' focus frequently results in considerable visual impairments, especially in accommodation under varying accommodative demands rapidly. This mechanism operates bidirectionally: the eye increases its optical power for focusing when transitioning from distant to near objects (accommodation) and decreases that power when shifting from near to distant objects (disaccommodation). The dynamic accommodation response is often characterized by four first order parameters: latency, peak velocity, response amplitude, and response duration. The latency measures the time delay between the stimulus onset and the response initiation (typically 300–400 ms), while the full response is completed in about 1 s (Del Águila-Carrasco et al., 2022; Suryakumar et al., 2007).

Accommodation response exhibits a dual-mode control mechanism characterized by fast and slow components (Hung and Ciuffreda, 1988; Khosroyani and Hung, 2002). The fast, open-loop component facilitates swift adjustments to step or rapid ramping stimuli. It is considered to be triggered by the blur stimuli when its amplitude and/or rate of change in velocity exceed certain threshold (Hung et al., 2002). The slow component, monitored by a closed-loop feedback mechanism, functions during steady-state or slow ramping stimuli, ensuring precise accuracy by continually refining the accommodation response (Hung and Ciuffreda, 1988; Khosroyani and Hung, 2002; Ciuffreda, 2002; Gamlin et al., 1994). Accommodative defecits observed in patients with mTBI might result from inappropriate function in both steady state and dynamic components. To fully understand the neurological impact of mTBI on accommodation control, it is critical to investigate both the rapid and gradual aspects.

Given the rapid and complex nature of dynamic accommodation responses, there is increasing interest in using objective measurement techniques to better characterize accommodative function in individuals with mTBI. Prior studies have reported consistent abnormalities in dynamic accommodation, including reduced amplitude, slowed peak velocity, and delayed response times in this population (Green et al., 2010; Thiagarajan and Ciuffreda, 2014; Haensel et al., 2024). However, this area of research remains relatively limited, particularly across diverse age groups, injury etiologies, and post-injury durations. These gaps underscore the need for further investigation using robust objective tools to better understand and monitor accommodative dysfunction in individuals with mTBI.

Understanding the extent and potential neural mechanisms underlying dynamic accommodation abnormalities in individuals with mTBI is essential for guiding clinical interventions. Neuro-optometric rehabilitation, including accommodative and oculomotor therapy, has been shown to improve visual function following mTBI (Thiagarajan and Ciuffreda, 2014). In addition, color filters (CFs) have been cli *Color filters (CFs) have been clinically applied to enhance* nically applied to enhance visual comfort in patients with TBI-related symptoms such as light sensitivity, visual stress, headaches and migraines (Tosta et al., 2024). These approaches reflect emerging strategies aimed at alleviating visual dysfunction and improving quality of life in affected individuals. In addition, color and luminance can influence the accommodation response in symptomatic individuals, with some colors potentially reducing accommodative demand and visual discomfort, while others may increase strain depending on the spectral properties and individual variability (Drew et al., 2012). Nonetheless, the precise processes by which these alterations influence accommodation response remain unclear. Notably, there is a gap in the literature regarding the objective effect of CFs on accommodation in individuals with mTBI (Fimreite et al., 2016).

It is still unknown whether application of clinical CFs directly impacts the accommodation system or works through other neurological pathways (Fimreite et al., 2016). This exploratory study addressed these gaps by measuring dynamic accommodation with subjectively selected CFs in both mTBI and control groups. This study posited that the mTBI group would demonstrate abnormal dynamic accommodation response compared to the control group. Additional, this study examined the impact of altering wavelength and/or luminance via different filters on dynamic accommodation parameters, contributing to understanding their therapeutic relevance in addressing visual dysfunction related to mTBI.

## Materials and methods

### Participants

A total of thirty-two participants with a medically diagnosed, self-reported history of mild traumatic brain injury (mTBI) within the past five years (ages 18–33) and sixty-four non-TBI control participants (ages 20 to 30) were recruited from the student body and local community of Pacific University. All participants provided written informed consent approved by the Institutional Review Board at Pacific University. The study followed the tenets of the Declaration of Helsinki.

All participants were required to be between 18 and 35 years old with best-corrected monocular distant visual acuity of at least 20/25. Residual refractive error after correction had to be ≤ 1.25 D hyperopia, ≤ 0.50 D myopia, ≤ 1.00 D astigmatism or anisometropia. Participants requiring refractive correction were instructed to wear habitual single-vision contact lenses throughout the study. None of the participants wore multifocal contact lenses or spectacles during the study.

Exclusion Criteria included any systemic or eye diseases, ocular trauma or surgery (including refractive surgery), history of amblyopia, neurological disorders, cognitive dysfunction, dyslexia, or previous treatment of the clinical binocular disorder. Participants who were taking medications that may affect accommodative function were also excluded. These include non-SSRI anti-anxiety drugs, anti-arrhythmic agents, anticholinergic, tri-cyclic antidepressants, or ADHA medications.

### Lens selection

The methods of lens selection has been described in previous publication from our laboratory (Almutairi et al., 2025). Breifly, the Intuitive Colorimeter Mk.3 (Cerium Visual Technologies, UK) was used to determine each participant's preferred lens color and optical density. Participants viewed high-contrast black text (12-point font) on a white background from 40 cm away. A forced-choice method compared no color to a specified color at saturation levels of 0 and 30, with hue adjusted in 30-degree increments. Participants rated visual comfort and text clarity, selecting the desired hue and saturation. The lens hue choice was determined based on a manufacturer-supplied program. PR-655/670 SpectraScan assessed luminance transmission and dominant wavelength. Each participant was also assigned a neutral density filter (ND) to match the photopic transmission of their selected color filter (CF). For supplementary materials of demographic data, selected lens profile and characteristics of the selected lens, please refer to Appendices A–D in Almutairi et al. (2025).

### Dynamic accommodation measurement

Dynamic accommodation response (accommodation and disaccommodation) was recorded from the right eye with an eccentric infrared photorefractor [PlusOptiX R09 - PowerRef 3 (PR3), Nuremberg, Germany]. PR3 is an open-field photorefraction capable of recording the accommodative dioptric power at a rate of 50 Hz with a resolution of 0.01 D (Plusoptix: PowerRef 3, 2023). Participants used a chin and forehead rest to reduce head movements throughout the experiment. The left eye was occluded using a long-pass infrared-transmitting filter (NEEWER^®^ 72 mm, 850 nm Infrared IR Pass Filter). This filter blocked all the visible wavelengths of light to the left eye while allowing infrared light from the PR3′s camera to pass through it to measure accommodation.

The dynamic response recording was done under moderately dimmed room lighting conditions, which remained constant throughout the experiment. The luminance level at the target position was 23 cd/m (Faul et al., 2010). Accommodation response was obtained with three testing filter conditions in a randomized sequence: (1) baseline without any lens (no filter, NF), (2) with the participant's preferred color filter (CF), and (3) with a neutral density filter (ND) that was approximately matched the luminance transmission of the chosen CF by the participants. The accommodation and disaccommodation targets were positioned at 20 cm (5 D, near target) and 6 m (~0 D, far target), respectively. The far target was viewed using a mirror placed at 2 m from the participant's eye. The target was a black letter E printed on white paper with high contrast. The size of both far and near targets is calibrated to subtend 2 min of arc visual angle on the retina (20/40 Snellen equivalent). Before testing, on-axis measurements were ensured by seating the participants appropriately and aligning them with the fixation targets. The participants were given one practice session to familiarize themselves with the experimental procedure.

Participants were asked to change their eye fixation alternately between the far and near targets every 15 s. The switching between the far and near targets was triggered by an audio command controlled by Experiment Builder (SR-Research, Ontario, Canada) (Alfaifi et al., 2019). The audio command was “far” for the 6 m target and “near” for the 25 cm target. Participants were instructed to respond to each audio command by changing their fixation between the targets as fast as possible and constantly maintaining the targets in focus. If the participant exhibited signs of inattention—such as failure to fixate on the target, noticeable gaze wandering, or delayed tracking response visible on the live monitor, the recording was interrupted. In such cases, the participant was reminded of the instructions, and the trial was repeated to ensure consistent data quality.

**Figure 1:**
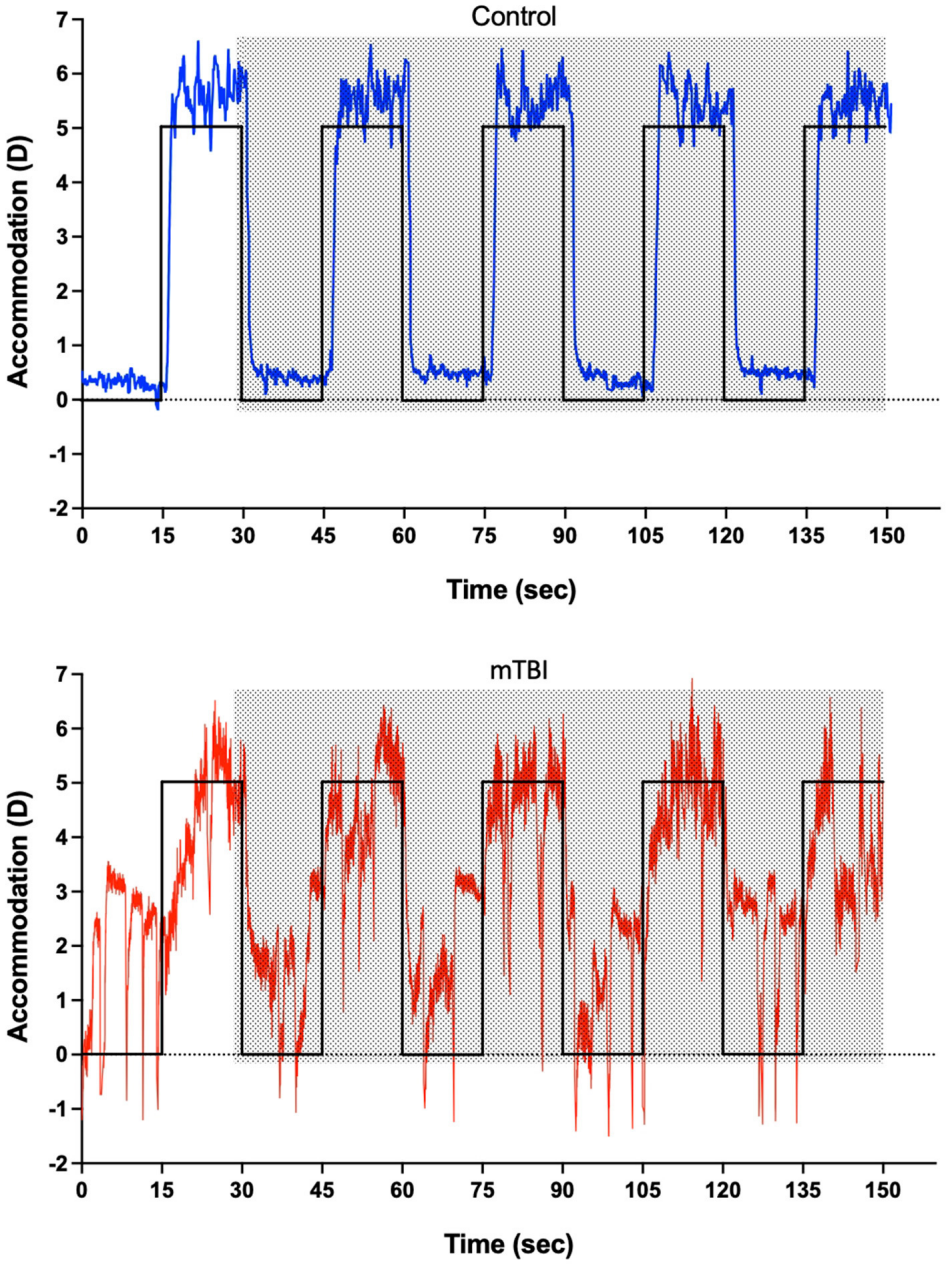
Representative raw data of accommodation response during monocular dynamic accommodation response without a lens (baseline). The black lines represent the near (5 D) and far (~0 D) stimulus levels. The **top graph** is the accommodation response trace from a normal control participant (N-11) (blue). The **bottom graph** is the accommodation response trace from a participant with mTBI (T-1) (red). The shaded area represents the four complete accommodation/disaccommodation cycles used for data analysis.

Repeated accommodation/disaccommodation cycles were recorded for 150 s for each lens testing condition. Each cycle lasted for 30 s, resulting in five cycles of response. The data was exported and divided into accommodation and disaccommodation (five recordings each). This repeated dynamic accommodation task was designed to simulate real-world near- far switching demands such as visual behavior in a classroom, as individuals with mTBI often experience difficulties with sustained or shifting accommodative effort in daily activities (Green et al., 2010). Participants started by looking at the far distance before the experiment began, and the experiment started with the audio command to look at far. Thus, no accommodation response was needed after the first command (far). The first cycle (accommodation/disaccommodation) was excluded to maintain equal data cycles for accommodation and disaccommodation responses. Only the last four complete cycles were included in the data analysis. Representative stimulus/response recording from a control participant and a mTBI participant is shown in Figure 1.

At the end of the experiment, the program automatically exited the experiment, and the investigator notified the participants to sit back and relax. Between each lens testing condition, participants were given a 5-min resting time, during which the investigator instilled an artificial tear in both eyes to minimize the possible eye dryness and fatigue.

### Data processing

To maintain data integrity, we limited the accommodation recording within ± 10 degrees of gaze position horizontally. It was shown that the accuracy of the recording is not affected by gaze position within 25 degrees temporal and 10 degrees nasally. Only the accommodation recordings when PR3 detected the pupil were used in the data analysis. The raw data was loaded into MATLAB (MathWorks 2019 software) for preparation, to remove artifacts, and to calculate the dynamic accommodation parameters. Blinks were removed from the data by identifying accommodative response measures that were 2 D away from the accommodative demand and replacing them using interpolation based on the research conducted by Hampson and Mallen (2012). The median of refraction for the NF condition at 6 m was considered a residual refractive error and subtracted from all other recordings to calculate dynamic accommodation.

### Dynamic accommodation and disaccommodation parameters analysis

Final position traces of accommodation and disaccommodation responses (units of diopters) obtained from the power refractor 3 were used for further dynamic analysis on MATLAB (Mathworks, Inc., MA, USA). The processing algorithm has been previously described by Labhishetty and Bobier (2017) and Bharadwaj and Schor (2005). Briefly, accommodation and velocity traces were smoothed over a 100 ms window. The initial point of the response occurred when the velocity surpassed 0.5 D/s, maintaining this level for the subsequent 100 ms. The end of the response was defined as the moment when velocity dropped below 90% of peak velocity and remained at that level for the subsequent 100 ms. The initial and final points determined through this criterion were subsequently validated through visual inspection. The opposite of this criterion was used for the disaccommodative responses.

### Data analysis

The data were analyzed using IBM SPSS Statistics 26 software. Mixed linear model was used to analyze the dynamic accommodation parameters: the latency, amplitude, peak velocity, and response time as dependent variables and the groups and filters as fixed factors. Pairwise comparison in the mixed linear model was used to test the difference in the dynamic accommodation parameters between groups and the effect of lens conditions within groups. We used Fisher's protected least squares tests, which balances alpha and beta errors by only considering unadjusted paired comparisons where the overall F for the main effect was significant. Graphing was performed using GraphPad Prism (GraphPad Software Inc., USA).

## Results

### Participants' demographics

Eight participants were excluded from the study according to the established inclusion and exclusion criteria. Five participants lacked their usual contact lenses during participation; one was amblyopic, and two had previously undergone vision therapy. Four additional participants were excluded due to PowerRe3′s limited capability to record from small pupil sizes and other technical difficulties. The data analysis included a total of 54 participants in the control group (age 21–30 years) and 30 participants with mTBI (age 18–33 years). There were 43 females and 11 males in the control group and 18 females and 12 males in the mTBI group. Some participants had missing data for some recording portions, resulting in varying participant numbers for different analyses.

### mTBI significantly impaired dynamic accommodative responses

**Figure 2:**
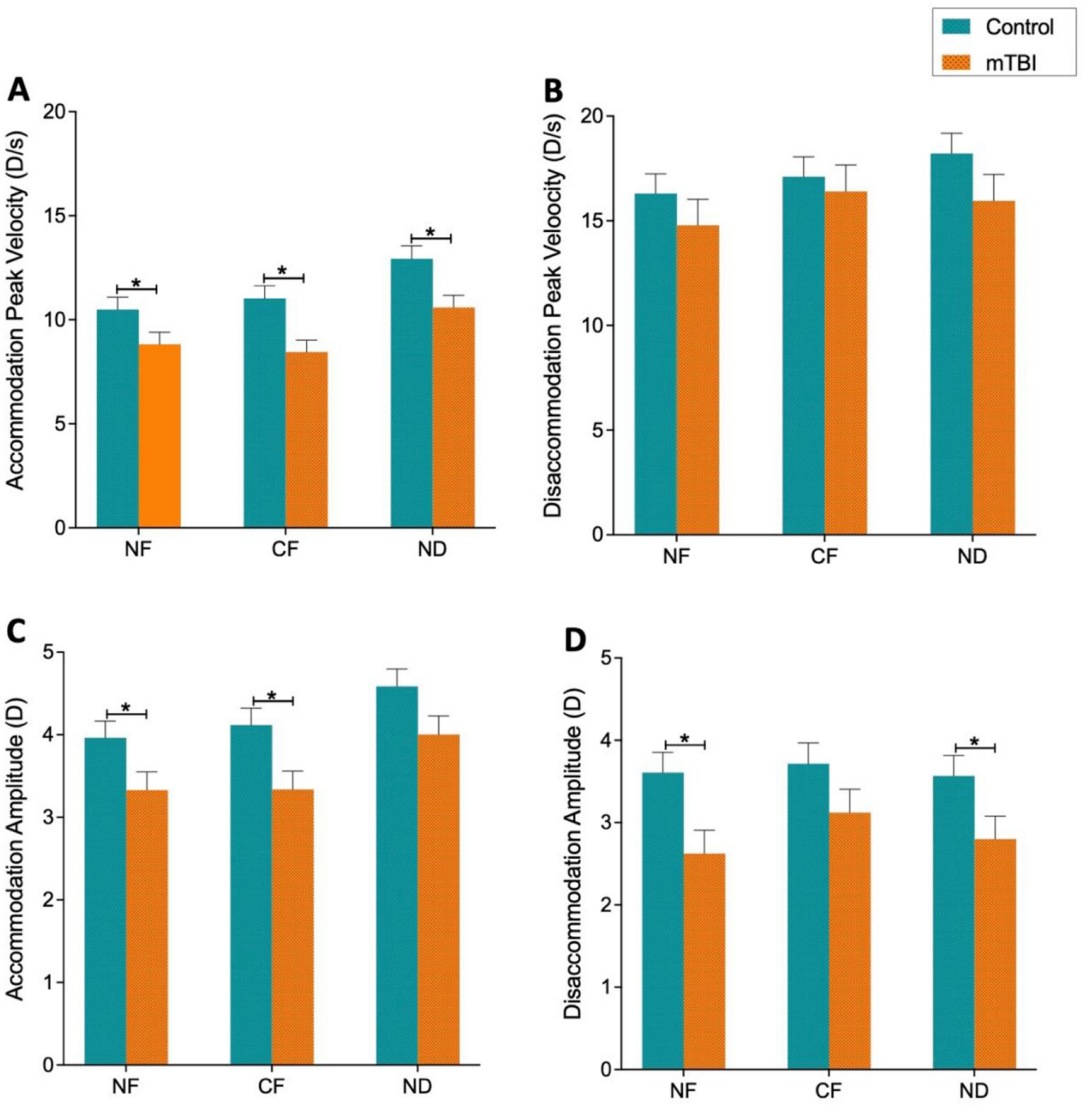
Between group comparison of mean values for accommodation and disaccommodation peak velocity **(A, B)** and amplitude **(C, D)** under three conditions: no filters (NF), color filters (CF), and neutral density filters (ND). Green bar represent control group, and orange bar represent mTBI group. Error bars represent the standard error of the mean. Asterisks denote statistically significant differences (*p* < 0.05).

At baseline (no filter, NF), mixed model analysis showed a significant main effect of group, indicating that the mTBI group exhibited significantly impaired dynamic accommodation performance compared to the control group. Specifically, accommodation peak velocity was significantly lower in the mTBI group [main effect: F_(1, 65)_ = 6.29, *p* = 0.01]. Accommodation response amplitude was also significantly reduced in the mTBI group relative to controls [main effect: F_(1, 64.7)_ = 4.6, *p* = 0.03], while there is no statistically significant changes observed in latency or response time (Figure 2 and Table 1). Similar impairment was also observed for disaccommodation amplitude at baseline, with the mTBI group demonstrating a significantly lower values than the control group [main effect: *F*_(1, 53.6)_ = 5.9, *p* = 0.018] (Figure 2 and Table 2). In contrast, no significant between-group differences were observed in disaccommodation peak velocity [F_(1, 57.3)_ = 1.14, *p* = 0.29], latency, or response time (Table 2). Since the latency and response time did not show any impairment induced by mTBI, these two parameters were no longer analyzed in the following sections.

**Table 1:** Dynamic accommodation parameter values, mean (SD), under different filter conditions in control and mTBI groups.

**Parameters**	**Baseline no filter**			**Color filter**			**Neutral density filter**		
	**Control**	**mTBI**	***P*** **value**	**Control**	**mTBI**	***P*** **value**	**Control**	**mTBI**	***P*** **value**
Amplitude	3.94 (1.54)	3.39 (1.24)	**0.035** ^*^	4.11 (1.55)	3.38 (1.17)	**0.019** ^*^	4.50 (1.50)	4.02 (1.01)	0.08
Peak velocity	10.37 (4.88)	8.54 (3.67)	**0.027** ^*^	10.72 (4.42)	8.37 (3.19)	**0.009** ^*^	12.37 (5.25)	10.02 (3.28)	**0.005** ^*^
Latency	0.35 (0.19)	0.35 (0.18)	0.84	0.36 (0.18)	0.37 (0.19)	0.90	0.33 (0.18)	0.32 (0.17)	0.82
Response time	1.67 (1.67)	1.46 (0.99)	0.18	1.65 (1.74)	1.49 (1.00)	0.39	1.73 (1.73)	1.50 (1.04)	0.79

Stars indicate between-group differnces at a significant level of p < 0.05. The significant *p* values are indicated in bold.

**Table 2:** Dynamic disaccommodation parameter values, mean (SD), under different filter conditions in control and mTBI groups.

**Parameters**	**Baseline no filter**			**Color filter**			**Neutral density filter**		
	**Control**	**mTBI**	***P*** **value**	**Control**	**mTBI**	***P*** **value**	**Control**	**mTBI**	***P*** **value**
Amplitude	2.83 (2.00)	2.14 (1.81)	**0.01** ^*^	2.93 (2.14)	2.72 (2.14)	0.12	2.93 2.13	2.50 (1.88)	**0.04** ^*^
Peak velocity	14.82 (8.16)	13.04 (7.50)	0.33	14.82 4.47	13.74 (8.95)	0.65	15.02 (8.24)	14.01 (8.22)	0.15
Latency	0.38 (0.46)	0.35 (0.42)	0.91	0.33 (0.46)	0.31 (0.43)	0.97	0.37 (0.43)	0.35 (0.46)	0.52
Response time	2.29 (2.50)	1.96 (2.71)	0.06	2.32 (2.77)	2.32 (2.77)	0.22	2.35 (2.99)	2.28 (3.17)	0.75

Stars indicate between-group differnces at a significant level of *p* < 0.05. The significant *p* values are indicated in bold.

### Partial rescuing effects of filters on mTBI-induced dynamic accommodation impairments

***Between-group comparisons demonstrated various partial rescuing effects by filters:*** mixed model analysis revealed a significant main effect of filters on the accommodation peak velocity [main effect: F_(2, 607.06)_ = 31, *p* < 0.01]. However, there was no interaction between group and filter [group^*^filter interaction: F_(2, 607.06)_ = 1.32, *p* = 0.26]. Similarly, a significant main effect of filter on accommodation response amplitude was also observed [main effect: F (2,605) = 29.8, *p* < 0.01], with no interaction between group and filter condition [group^*^filter interaction: F_(2, 605)_ = 0.62, *p* = 0.58]. This indicated that the filters had comparable directional effects across both groups for these parameters.

Between group comparison in *post-hoc* test showed that for the mTBI-induced impairment, the significant reduction in the accommodation peak velocity persisted under CF and NF conditions (Figure 2A, Table 1). However, filter conditions seemed to have differential effect on response amplitude for accommodation and disaccommodation. For the accommodation amplitude, the impairment demonstrated by the group difference remained significant under the CF, but was no longer statistically significant under the ND condition (Figure 2C, Table 1). This might suggest a potential normalizing effect of the ND filter on this specific parameter. In contrast, interestingly, such rescue effect was observed for disaccommodation amplitude under CF, not the ND condition (Figure 2D, Table 2).

This finding supports the interpretation that filters may restore aspects of dynamic accommodation function in individuals with mTBI to levels comparable to controls.

**Figure 3:**
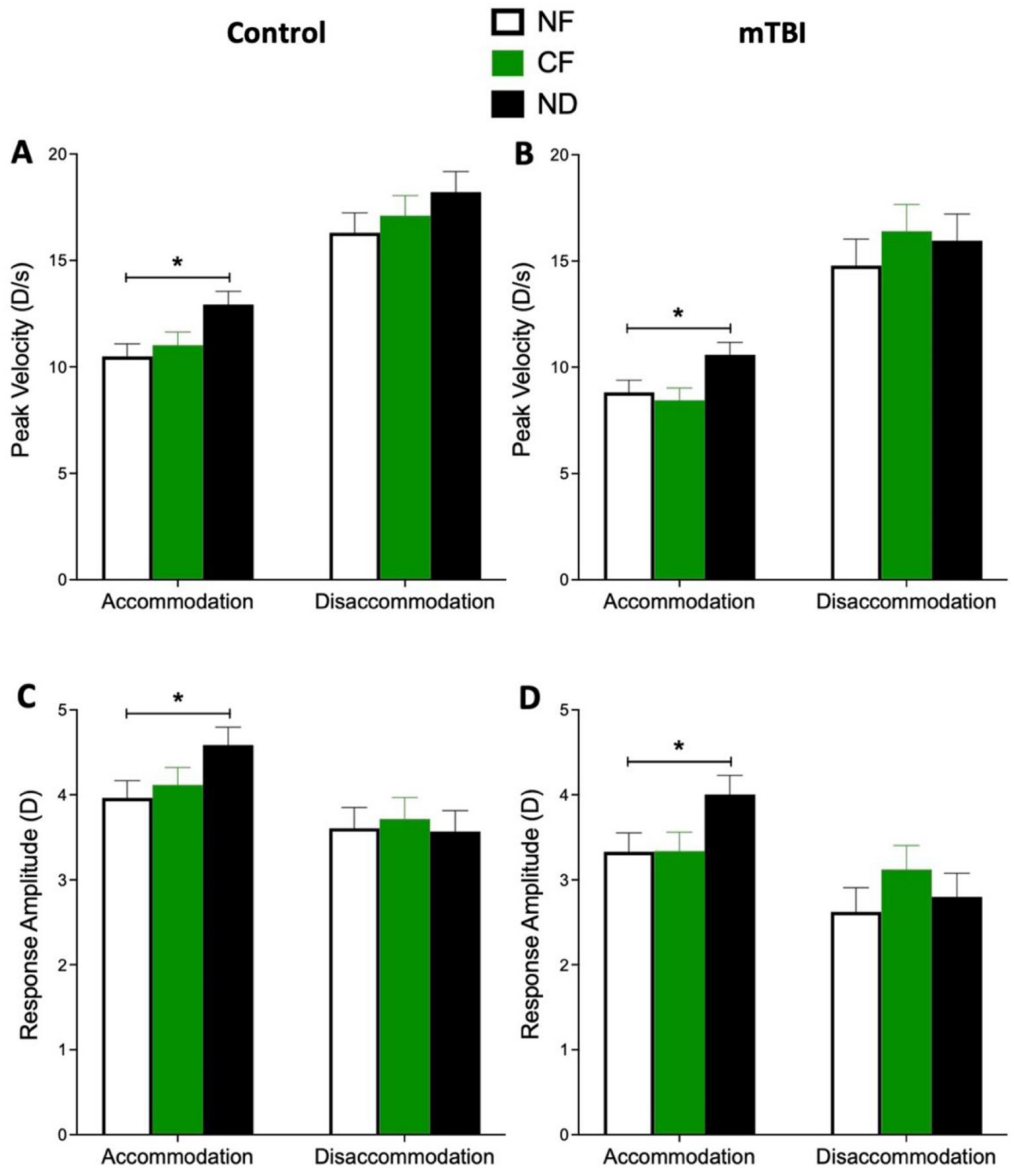
Within group comparison for the mean of the accommodation and disaccommodation peak velocity and amplitude under no filters (NF), color filters (CF), and neutral density (ND) conditions for the control **(A, C)** and mTBI **(B, D)** groups. The error bars represent the standard error of the mean. Asterisks indicate a significance level of *p* < 0.05.

***Within group comparisons highlighted partial rescuing effects by ND filters:*** In the mTBI group, filter condition had a significant effect on accommodation peak velocity [F_(2, 271)_ = 13.8, *p* < 0.001]. *Post hoc* tests revealed that while the color filter (CF) did not significantly differ from the baseline (NF) condition (*p* = 0.37), the neutral density (ND) filter significantly increased peak velocity compared to baseline (mean difference = 1.77 D/s, *p* < 0.001) (Figure 3B). This indicated that the ND filter restored peak velocity to a level comparable to the control group at baseline.

Accommodation amplitude was also significantly influenced by filter condition in the mTBI group [F_(2, 214.5)_ = 18.6, *p* < 0.001]. Again, CF did not exert significant change from NF condition (*p* = 0.94). However, ND significantly increased accommodation amplitude from baseline [mean difference = 0.67 D, *p* < 0.001] (Figure 3D). This finding reinforces the potential rescuing effect of ND filters in individuals with mTBI.

Disaccommodation parameters, including peak velocity [F_(2, 216.8)_ = 1.6, *p* = 0.20] and amplitude [F_(2, 152.2)_ = 1.9, *p* = 0.14], were not significantly affected by filter condition within the mTBI group (Figures 3B, D).

Similar pattern of effect was also observed in the control group. There was a significant main effect of filter condition on accommodation peak velocity [F_(2, 391.1)_ = 29.1, *p* < 0.001]. *Post hoc* analysis revealed no significant difference between CF and NF (*p* = 0.13). In contrast, the ND filter significantly increased peak velocity compared to NF (mean difference = 1.96 D/sec, *p* < 0.001) (Figure 3A). Accommodation amplitude was similarly affected by filter condition [F_(2, 390.5)_ = 14.9, *p* < 0.001]. ND resulted in a significant increase in the amplitude (mean difference = 0.62 D, *p* < 0.001), while CF showed no significant effect (*p* = 0.16) (Figure 3C). There were no significant filter effects on disaccommodation peak velocity [F_(2, 333)_ = 2.8, *p* = 0.06] or disaccommodation amplitude [F_(2, 217.6)_ = 0.17, *p* = 0.83] in the control group (Figures 3A, C).

## Discussion

This study aimed to investigate dynamic accommodation performance in individuals with mild traumatic brain injury (mTBI), and to evaluate the effects of two optical interventions, color filters (CFs) and neutral density (ND) filters, on key accommodative parameters. Given that accommodative dysfunction is a common cause for visual complaint in mTBI, and that filter lens are often prescribed for symptom relief, this study sought to objectively quantify their impact on dynamic accommodation. Our findings revealed two primary outcomes. First, at baseline (no filter), individuals with mTBI exhibited significantly reduced accommodation and disaccommodation amplitude and peak velocity compared to healthy controls, consistent with prior reports of accommodative deficits in this population. Second, and more importantly, application of ND filters significantly improved both accommodation amplitude and velocity in the mTBI group, effectively restoring their performance to near control levels. In contrast, subjectively selected CFs seemed to have mixed effect: between-group analysis indicated a potential rescuing effect for disaccommodation amplitude, while within-group comparison showed no significant effect on any dynamic accommodation parameter. These results underscore the potential rehabilitative utility of ND filters in addressing accommodative deficits following mTBI, while highlighted urgent need for further investigation for CF.

These findings have important clinical implications for visual rehabilitation in individuals with mTBI. The ability of ND filters to enhance accommodation amplitude and velocity suggests they may serve as a non-invasive, cost-effective adjunct to traditional neuro-optometric therapy. While color filters are often prescribed based on subjective comfort, our results indicate that luminance reduction via ND filters leads to more concrete objective improvements in accommodative performance. This supports their inclusion in optometric management plans, particularly for patients exhibiting slowed or reduced accommodative responses. Future studies should explore whether ND filters improve real-world visual tasks (e.g., reading, screen use) and whether their benefits are sustained with prolonged use.

The neural control of accommodation is described by the dual-mode model, which includes a fast, open-loop component and a slower, closed-loop feedback mechanism (Hung and Ciuffreda, 1988; Khosroyani and Hung, 2002; Hung and Semmlow, 1980). The fast component is preprogrammed and responsible for initiating a rapid “pulse” response that accounts for most of the stimulus demand, particularly in response to sudden blur changes. This mode is typically triggered by step or fast-ramping stimuli, defined as stimuli with a rate exceeding 0.5 D/s (Hung et al., 2002). In contrast, the slow component is driven by feedback mechanisms that help fine-tune the response to reach and maintain target focus. The velocity of accommodation under fast-mode control follows the main sequence relationship, whereby greater stimulus amplitudes produce faster peak velocities (Schor and Bharadwaj, 2006). Our study used a 5.00 D step stimulus, which exceeds the fast-mode threshold, thereby predominantly activating this component. The observed reduction in amplitude and peak velocity in the mTBI group may therefore reflect impaired functioning of this fast mechanism. Notably, response latency remained unaffected in the mTBI group, which aligns with prior reports that latency during fast-mode responses is relatively fixed and independent of stimulus velocity (Hung and Ciuffreda, 1988; Khosroyani and Hung, 2002; Hung and Ciuffreda, 2002). This preservation of latency suggests that the initial triggering of the accommodative response remains intact in mTBI, while the velocity- and amplitude-generating mechanisms downstream may be selectively disrupted. In more severe TBI cases, latency abnormalities might emerge.

The accommodation responses in the mTBI group were characterized by reduced amplitude and slowed velocity compared to controls, consistent with previous reports of impaired accommodative dynamics following brain injury (Green et al., 2010; Thiagarajan and Ciuffreda, 2014; Haensel et al., 2024). Visual inspection of several response traces in the mTBI group suggested increased variability and possible stepwise progression in the accommodative response. However, these patterns were not formally analyzed in the current study. Future work should investigate temporal irregularities or non-linear trajectories using trial-level signal variability or noise modeling to better characterize response dynamics in mTBI.

Due to the complexity of the neural pathways involved in accommodation, diffuse axonal injury following brain trauma can adversely affect cortical and subcortical structures responsible for generating accommodative responses (Thiagarajan and Ciuffreda, 2022; Ciuffreda and Thiagarajan, 2022). One such structure is the Edinger–Westphal (EW) nucleus located in the midbrain, which sends parasympathetic output to the ciliary muscle via the oculomotor nerve. Animal studies have shown that different populations of neurons within the EW nucleus respond selectively to accommodation stimuli and encode key dynamic parameters such as response velocity and amplitude (Gamlin et al., 1994; Mays and Gamlin, 2000; Thiagarajan and Ciuffreda, 2022; Ciuffreda and Thiagarajan, 2022). Given its integrative role, damage to the EW nucleus or its cortical projections may impair the encoding of motor output signals required for rapid accommodative changes. Additionally, the brainstem, where the oculomotor nuclei reside, and the fiber tracts connecting it to supranuclear control centers are particularly susceptible to mechanical strain during rapid head movements, such as rotational injuries common in mTBI (Rucker et al., 2019).

In the current study, CFs did not show consistent influence on the dynamic accommodation parameters during accommodation and disaccommodation. Interestingly, the ND filter enhanced both the amplitude and peak velocity of accommodation, but not disaccommodation, in both groups. This suggests that ND filters may exert a rescuing effect restoring accommodative performance in the mTBI group to near normal (control baseline) levels. One possible explanation for the enhanced accommodative performance with ND filters relates to the preservation of longitudinal chromatic aberration (LCA) cues. LCA is a natural optical phenomenon in which different wavelengths of light focus at different depths in the eye, with short wavelengths (blue) focusing in front of long wavelengths (red). The visual system uses this chromatic difference as a cue to interprete the direction of the retinal blur signal and to guide accurate focusing (Kruger et al., 1997; Cholewiak et al., 2018). ND filters uniformly reduce the intensity of all wavelengths, thereby preserving the spectral balance and LCA cues. In contrast, color filters (CFs) selectively reduce portions of the visible spectrum, potentially weakening or distorting the LCA signal. Prior studies have shown that removing or reversing LCA, such as through monochromatic or narrow bandwidth light, can impair accommodation performance (Kruger et al., 2000). While we did not directly test this mechanism, the relative efficacy of ND filters in our study may reflect their ability to preserve more natural visual input, including chromatic signals.

### Limitations

This study has several limitations. First, the 5-minute dark adaptation and recordings conducted under dim room lighting may have created a significant contrast with the dark environment due to the bright computer screen, potentially overwhelming a dark-adapted visual system. Excessive light may worsen visual discomfort symptoms, especially in participants with mTBI. Second, the ND did not precisely match the luminance transmission of CF due to a significant step in optical density. When the CF's transmission was between steps, we systematically selected ND matching to the higher step. As a result, the ND decreased the overall luminance more than the CF. This may explain the stronger effect of the ND on participants' accommodative response compared to CF. Future experiments should consider reversing the polarity of the stimulus presentation on screen and calibrating the surrounding lighting conditions (Almutairi et al., 2025). Third, The monocular visual task does not simulate a natural viewing condition. This study focused solely on the accommodative system; however, the exclusion of vergence input may influence the accommodative response. Both accommodation and vergence systems are frequently compromised in mild traumatic brain injury (mTBI) (Merezhinskaya et al., 2019). Fourth, the color filters used in this study were selected based on near viewing (40 cm), whereas the dynamic accommodation task involved viewing a target at 6 m. It is possible that filters optimized for near vision may not yield the same effects for distance-to-near transitions, which could have contributed to the lack of measurable effect of CFs on accommodative performance. Finally, although the repeated accommodative cycles were designed to simulate real-world dynamic visual demands rather than to intentionally induce fatigue, it is possible that cumulative effort may have contributed to performance variability over time. However, we did not analyze trial-by-trial changes to directly assess fatigue effects. Future studies may consider including temporal analyses to better understand potential fatigue-related impacts.

### Conclusion

This study found that individuals with mTBI exhibited abnormal velocity and amplitude in dynamic accommodation, reflecting slowed and weakened accommodative responses at baseline. Importantly, the use of neutral density (ND) filters significantly improved these parameters in the mTBI group, restoring their dynamic accommodation performance to near control levels. In contrast, subjectively selected color filters (CFs) did not produce consistent effect. These findings highlight the potential clinical utility of ND filters as a non-invasive adjunct to neuro-optometric rehabilitation. Future research should explore the effects of ND filters in real-world tasks and extend this work to include moderate and severe TBI, as well as other accommodative paradigms such as ramp or sinusoidal stimuli.

## Data Availability

The raw data supporting the conclusions of this article will be made available by the authors, without undue reservation.
